# Death of M. Orfila

**Published:** 1853-06

**Authors:** 


M. Orfila, the eminent physician and toxicologist, died in Paris on Saturday, the 12th of March, of pneumonia, after a short illness, aged seventy. He had lectured on the 4th in comparative health and spirits, before as numerous an audience as had assembled around him for the last thirty-four years. The funeral obsequies of M. Orfila were performed on the 15th of March last with great pomp, at the church of St. Sulpice. Nearly the whole of the 20th batallion of the National Guard was present to render military honors to the deceased, as grand officer to the Legion of Honor. The church was crowded, and amongst the congregation were the greater part of the members of the Faculty of Medicine, the Academy of Medicine, and the Academy of Sciences. The eminent men of science never met in greater numbers than on this occasion. Many of the high public functionaries were also present. The cords of the pall were held by MM. P. Dubois, Berard, Dubois d Amiens and De Bussy. After the religious service, the body was conveyed to the cemetery of Mount Parnasse, followed by an immense line of mourners.

M. Orfila was by birth a Spaniard, but was naturalized in France in the early part of the reign of Louis Philippe. In 1805, M. Orfila went to sea in a merchant vessel, and it was intended by his friends that he should enter the navy, but he had already a strong inclination for the Medical profession, and suddenly abandoned the sea and went to Valencia to study medicine. As a student he greatly distinguished himself, and carried off the first prize in physics and chemistry. A favorable report having been made of his studies to the Junta of Carcelona, that body resolved to send him to Paris, to study the natural sciences, and a sum of 1500f. per annum was voted to him for that purpose. He went to Paris in 1807, and had hardly been there ten months when war broke out between France and Spain. He was thus deprived of pecuniary resources for continuing his studies, but he had fortunately an uncle established at Marseilles, who agreed to provide him with 1500f. per annum, until he should obtain the diploma of Doctor in Medicine, when this allowance was to cease. M. Orfila passed a brilliant examination, and obtained his diploma. Having no longer any funds at his command, he opened a course of lectures in chemistry, which was well attended and furnished him with the means of living. Some of the most eminent men of the present day were among his pupils: among them may be mentioned Jules Cloquet, Beclard, sen., and M. Edwards. The reputation of M. Orfila continued to increase, and, in 1816, he was appointed one of the physicians of Louis XVIII. He was next elected a Professor of the Faculty of Legal Medicine, and in 1823 he was chosen to fill the chair of Chemistry. He had already been elected a member of the Academy of Medicine. The Revolution of 1830 opened to M. Orfila a new era of distinctions. He was successively elected Dean of the Faculty, member of the Council-General of Hospitals, and member of the Council General of the Department. After he had received his letters of naturalization, he was appointed a member of the Council of Public Instruction, and was successively named chevalier, and officer and commander of the Legion of Honor. Most readers are aware of the importance attached to the opinions of M. Orfila, in cases of legal medicine. In cases of poisoning, where much depended on the medical evidence he was invariably called upon by the Courts of Assize. His evidence in the Lafarge case and others created great sensation, and had a decided influence on the juries. The scientific reputation of M. Orfila may be said to have commenced with his *Treatise on Poisons; or General Toxicology.* The next works published by him, which acquired European reputation, were the *Elements of Legal Medicine* and *Lessons on Legal Medicine,* which went through several editions; he was also the author of many other works of almost equal celebrity. We may also mention the publication, some years ago, of his Lectures on Organic Chemistry. During the whole reign of Louis Philippe, M. Orfila remained at the head of the Faculty of Medicine, but after the revolution of February, the Provisional Government revoked his functions. In the course of his researches into the action of poisons, M. Orfila performed numerous experiments with animals, some of which, from the great suffering that they occasioned, induced a belief among persons who were not personally acquainted with him, that his nature was cruel. This, however, was far from being the case. He was humane and benevolent, and it was with great pain to himself that he was compelled, in order to make science useful to his fellow-creatures, to perform some of these experiments. In the celebrated Lafarge case, M. Raspail, who was opposed to him, disputed with great energy most of his statements, but without effect; and subsequently the opinion expressed by M. Orfila, in opposition to that of M. Raspail, as to the absorption of poisons by the human body after interment, by contact with the earth, to such an extent as to reveal the presence of a quantity which would lead to a supposition that it had been administered during life, has been confirmed by most of the eminent men who have been examined on such questions before Courts of Assize. M. Orfila suffered physically for some time before his death, and had been long a severe mental sufferer from the affliction caused by the illness of his son, who had become epileptic, and so affected in mind that it was found necessary to place him in a maison de santé.

M. Orfila has bequeathed a large scientific Museum to the town of Angers, a place for which he entertained a great affection; and he has also left 120,000f. to the Academy of Medicine, to found scientific prizes.—*Lancet and Med. Times and Gazette.—Med. News.*

